# Arvind Kumar Baronia: A Visionary Indian Academician in Critical Care Medicine

**DOI:** 10.7759/cureus.70250

**Published:** 2024-09-26

**Authors:** Mohan Gurjar, Afzal Azim

**Affiliations:** 1 Critical Care Medicine, Sanjay Gandhi Postgraduate Institute of Medical Sciences, Lucknow, IND

**Keywords:** academics, critical care medicine, historical vignette, india, intensive care, medical education, teaching and training

## Abstract

Dr. Arvind Kumar Baronia, an esteemed academician, devoted his professional life to laying the foundation of the specialty of critical care medicine (CCM) in India in the public sector. His vision and efforts to provide world-class clinical care to patients requiring intensive care units not only led to the establishment of the first standalone department of CCM in the public sector in India but also contributed significantly to formulating a well-structured training course for CCM as well as to get recognition of this specialty as a super-specialty branch of medicine in India. These academic milestones ultimately continue to create a pool of well-trained critical care physicians from various teaching institutes and colleges in India who serve to save thousands of lives of critically ill patients throughout the country.

## Introduction and background

At the beginning of the 21st century, the critical care medicine (CCM) specialty became an established branch of medicine in most of the developed countries [1-3] but was in its infancy in India. In that era, most of the intensive care units (ICUs) in the country were "open ICUs," which means that critically ill patients were admitted under the direct care of physicians or surgeons, and anesthesiologists were supposed to provide ventilator support only [4]. In fact, there were no known trained critical care specialists, such as "intensivists" or "critical care physicians," to manage ICU patients. In 1993, a professional society "The Indian Society of Critical Care Medicine (ISCCM)" was formed and, later on, started a one-year training course in hospitals of the non-public sector [5]. However, there was not much availability of dedicated critical care services in the public sector hospitals in India.

Dr. Arvind Kumar Baronia played a pivotal role in the academic journey of CCM within the public sector in India, yet his contributions remain underappreciated in the broader history of the specialty’s development in the country [4]. His visionary leadership and dedication were instrumental in establishing the first standalone department of CCM in the public sector in India [6,7]. Soon after, he realized the importance of a well-structured training course in the specialty of CCM and its approval by the national regulator, the Medical Council of India (MCI; now known as the National Medical Commission (NMC)). In 2010, his tireless efforts with justification and representation through the competent authorities laid the foundation for the recognition of the specialty of CCM as a super-specialty branch of medicine in India [8]. These milestones helped cultivate a generation of trained critical care physicians, many of whom now serve in leading capacities across the nation. His work continues to influence the practice and academic growth of CCM in India. This article collates the information from published literature, institutional websites, and departmental archives to seek to shed light on Dr. Baronia’s unparalleled contributions to CCM in India, celebrating his visionary academic and clinical leadership that shaped the specialty in the public sector.

## Review

Dr. Baronia’s life and career

**Figure 1:**
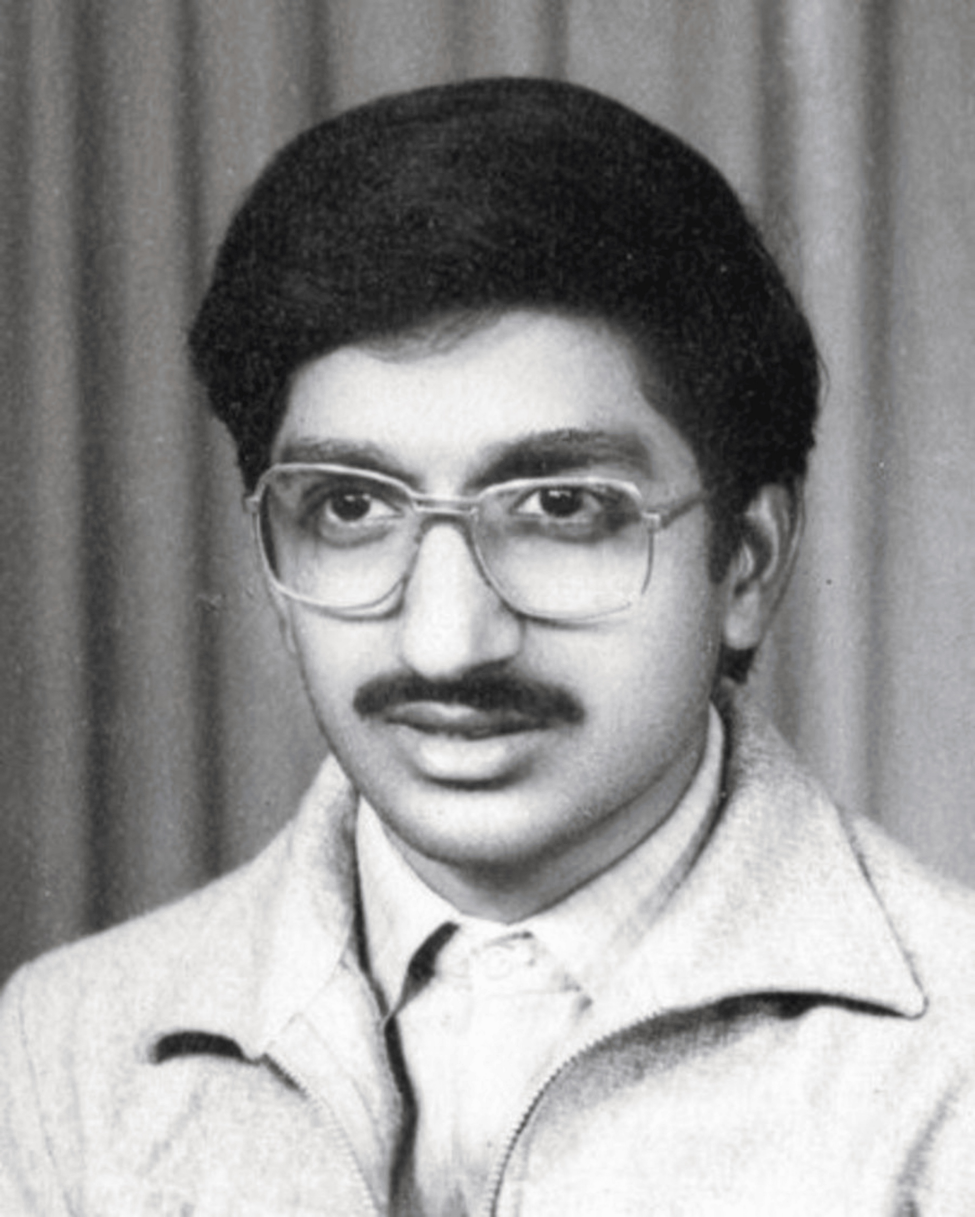
Dr. Arvind Kumar Baronia during his initial career Permission to publish this photograph has been obtained from Dr. Arvind Kumar Baronia.

Born in 1959, Dr. Baronia received his early education in the city of Jhansi, located in the Indian state of Uttar Pradesh. Dr. Baronia (Figure 1) received his MBBS degree (1978-1983) from King George’s Medical College, Lucknow (India), and then an MD anesthesiology degree (1984-1988). Thereafter, he received four years of training as a senior registrar in some of the high-ranking teaching institutions of the country, before joining Sanjay Gandhi Postgraduate Institute of Medical Sciences (SGPGIMS), Lucknow, as an assistant professor in the department of anesthesiology in 1991. Because of his immense interest in serving critically ill patients, Dr. Baronia enriched his experience in critical care by working as a full-time registrar at Belfast City Hospital under the overseas clinical fellowship program of Northern Ireland from 1994 to 1995. At SGPGIMS, through departmental promotions, he transitioned from assistant to associate professor followed by additional professor till 2002 when he left anesthesiology as he got an offer to serve as head of the newly created CCM department in the same institute [6]. Dr. Baronia continued to steer this new specialty for almost two decades as the founder and head of the department. He took voluntary retirement from the institute in the year 2020 and then served the Government Medical College in the remote Himalayan city of Pithoragargh. Currently, he is living in a small hamlet, Naukuchiyatal, in the Nainital district of Uttarakhand, a state in India.

Establishment of the first standalone academic department of CCM in India

SGPGIMS, being a tertiary care super-specialty medical institution situated in north India, caters to patients from a large geographical area not only from the states of Uttar Pradesh, Bihar, Odisha, and West Bengal but also from neighboring countries like Nepal and Bangladesh [9]. The patients are referred to SGPGIMS from other hospitals on account of either diagnostic issues or inability to handle the severe and complex nature of the illness, and a large number of these patients need critical care services during treatment. Dr. Baronia took advantage of the legacy of SGPGIMS to establish a new department, CCM, as this premier teaching institute has a glorious history of being the first in the country to introduce new specialties such as clinical immunology, medical genetics, endocrine surgery, transfusion medicine, nuclear medicine, and more [10]. Dr. Baronia organized a brainstorming session in July 2000 on the need for a dedicated CCM department by inviting leading critical care physicians across the country [6]. During this brainstorming session, leaders agreed that critical care is best delivered as a "closed model" by a team consisting of full-time critical care physicians. Also, having a general ICU rather than separate sub-specialized ICUs (medical, surgical, respiratory, etc.) allows concentration and encourages efficient use of skills and resources.

After this national-level brainstorming session and approval from the competent authorities, the department came into existence in September 2002 and became the first standalone academic department of CCM in the public sector in India [6,7]. Initially, the department started its clinical services with eight beds in the level-3 ICU, which means each bed had a facility for advanced invasive monitoring and availability of organ-support equipment. Since the beginning, the department followed the "closed ICU" model for a timely and better decision-making process in the very dynamic nature of the clinical condition of ICU patients.

At that time, there were various training models worldwide for becoming a critical care physician, including multidisciplinary super-specialty, multiple sub-specialty, single-based specialty, and primary specialty programs. Dr. Baronia adopted a multidisciplinary super-specialty model, where trainees have access to a common national core curriculum for CCM after completing their post-graduation in the primary specialty. The department allowed clinical training in CCM after a national-level competitive examination process, once trainees completed their post-graduation either in anesthesiology, internal medicine, or pulmonary medicine. Dr. Baronia prepared two separate critical care manuals each for doctor trainees and nursing staff to standardize teaching, training, and clinical care in the department.

Dr. Baronia introduced highly skilled procedures like percutaneous dilatation tracheostomy both in adult and pediatric age groups, prone positing for acute respiratory distress syndrome, and pulmonary artery catheterization in indicated ICU patients. These skilled procedures not only helped in the management of patients, but trainees also got learning experience while assisting him. Dr. Baronia took a leadership role in the institute by starting departmental outreach services for any critically ill patients being managed by other specialties in the ward. The outreach services had a broader scope, including but not limited to timely resuscitation, nutrition advice, dyselectrolytemia management, antimicrobial optimization, etc.

Dr. Baronia knew the importance of the academic program for the sustained progress of the specialty. He framed and started a one-year training program in CCM in 2005 so that trainees get academic recognition in the country [6]. This one-year post-doctoral certificate course (PDCC) in CCM had two seats per year, and trainees, selected through a national merit-based entrance examination, had to pass an exit cum qualifying examination at the end of their training [6,11]. Over time, the number of candidates appearing for entry-level exams increased significantly, and PDCC seats were increased from two to four per year in 2010 [6,11].

Contribution to get recognition of CCM as a super-specialty in India

Dr. Baronia knew that there was a complete void in all aspects of trained CCM specialists, including teaching, training, and research, to meet the demands of a vast country, like India. He also knew that the MCI (now known as the NMC), which is the highest national regulatory body in the field of medical education, should recognize CCM as a super-specialty. He approached the MCI in 2010 through the competent authority of the institute with his detailed documentation about the justification for the need to get recognition of CCM as a super-specialty with a three-year training program. Later on, in the year 2010, MCI recognized CCM as a super-specialty course [8]. Dr. Baronia also undertook the responsibility to formulate the first curriculum of the three-year training course Doctorate of Medicine (DM) in CCM as part of the committee formed by MCI [12].

The department started a three-year training program (DM) in CCM in 2013 after recognition from the MCI [6,7]. At present, a three-year DM in CCM training course is running successfully at almost 15 public sector institutes in India [13]. Currently, there are two national merit-based entrance examinations, the Institute of National Importance Super Specialty (INI-SS) and the National Eligibility Cum Entrance Test Super Specialty (NEET SS), being conducted to get admission in either the Institute of National Importance or other public sector academic institutes, respectively [14,15].

Large pool of CCM-trained physicians from the department

**Figure 2:**
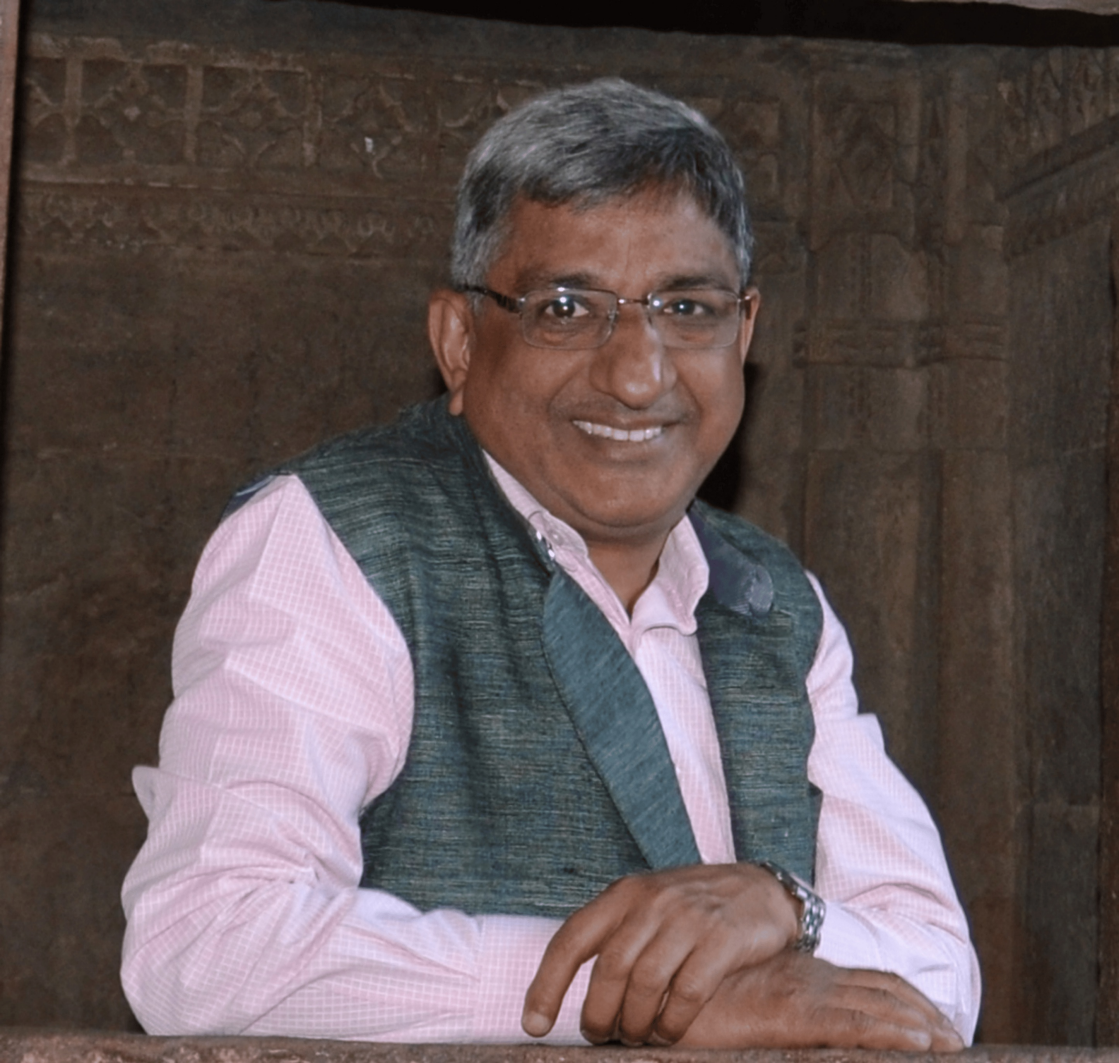
Dr. Arvind Kumar Baronia as head of the Department of Critical Care Medicine at Sanjay Gandhi Postgraduate Institute of Medical Sciences, India Permission to publish this photograph has been obtained from Prof. Banani Poddar.

Dr. Baronia sowed the seed by establishing the CCM department, which is now giving the fruits in the form of trained critical care physicians. Over the last two decades, more than 150 trainees have been trained in CCM, including 56 in the one-year program PDCC and 19 in the three-year program DM course [6]. During the detailed clinical rounds, Dr. Baronia (Figure 2) used to ignite the young minds of trainees for a better understanding of the complex and dynamic disease process of ICU patients [16]. These trained physicians are now serving and leading the CCM specialty throughout the country, including premier institutions, medical colleges, defense services, district hospitals, and non-public sector hospitals [17].

Vision for a center of excellence for research in the field of CCM

Dr. Baronia had created a conducive environment for doing high-impact research by faculty and trainees for the benefit of critically ill patients at large. Over the last two decades, the department attained the position of a flag bearer in the field of research and publication in the specialty of CCM from India. The department has published more than 300 research papers and received funding and support for collaborative research and patent grants for innovative devices [18,19].

## Conclusions

Dr. Baronia’s multifaceted contribution to the CCM specialty in India as a founder academician, teacher, thinker, administrator, and hard-working clinician will be cherished by his trainees, who now become trainers throughout the country.
